# Editorial: Targeting innate and adaptive immunity for improvement of cardiovascular disease

**DOI:** 10.3389/fphys.2022.952837

**Published:** 2022-08-11

**Authors:** Henrike Janssen, Nana Maria Wagner, L. Christian Napp, Jan Larmann

**Affiliations:** ^1^ Department of Anesthesiology, Heidelberg University Hospital, Heidelberg, Germany; ^2^ Department of Anesthesiology, Intensive Care Medicine and Pain Therapy, University Hospital Muenster, Muenster, Germany; ^3^ Department of Cardiology and Angiology, Hannover Medical School, Hannover, Germany

**Keywords:** immunity, inflammation, cardiovascular disease, leukocytes, atherosclerosis, immune dysfunction

According to the WHO’s Global Health Estimates 2019, the deleterious complications of cardiovascular disease, in particular ischemic heart disease and stroke, account for 27% of deaths worldwide, rendering it the single most detrimental disease globally.

Extensive effort has been put into preventive interventions, such as anti-smoking campaigns, educational programs for dietary changes and exercise to improve overall cardiovascular health and prevent development of cardiovascular risk factors already in preschool students (Gupta and Wood, 2019; Santos- Beneit et al., 2022).

Pharmacological interventions so far mainly focused on modulation of blood pressure, hyperlipidemia and glucose metabolism, as well as antithrombotic therapy, but further optimization has stalled (Arnett et al., 20192019; Fang et al., 2021).

Atherosclerosis is the leading underlying pathology of cardiovascular disease, and shares many features with other chronic inflammatory conditions. The concept of bone marrow derived macrophages destabilizing atherosclerotic lesions has been widely accepted, and recent scientific findings have broadened our understanding on how every part of the immune system plays a role in vascular inflammation (Chinetti-Gbaguidi et al., 2015; Engelen et al., 2022) and how stressors such as perioperative inflammation impact on atherosclerosis (Janssen et al., 2015; Janssen et al., 2020).

The CANTOS trial, published in 2017, was the first to prove that an anti-inflammatory therapy by targeting interleukin-1β with a monoclonal antibody is sufficient to reduce recurrence of cardiovascular events (Ridker et al., 2017). This breakthrough was followed by promising interventions with colchicine in 2019 and 2020 (Tardi f et al., 2019; Nidorf et al., 2020). These studies have given hope to patients with severe cardiovascular disease who have suffered cardiovascular events despite all other efforts.

This Research Topic addresses the current state-of-the art in innate and adaptive immunity with its implications for cardiovascular complications. Comprehensive review articles and extensive original research studies broaden our view on organ crosstalk in vascular disease, and explore therapeutic options.

In a narrative review, Sauter et al. report on the role of macrophages, dendritic cells and platelets in advanced atherosclerosis. Kaiser and colleagues build-up on this work, giving unique and in-depth insights on platelet-neutrophil crosstalk. They address mechanisms of immune and immunothrombotic responses, describe the involvement in different cardiovascular conditions such as atherosclerosis, thrombosis, ischemia-reperfusion injury and COVID-19-related pathology and depict recent therapeutic approaches. Funk-Hilsdorf and colleagues genuinely summarize the present knowledge on the role of innate and adaptive immune responses in pathogenesis and disease progression of pulmonary hypertension. By dissecting the contribution of various immune cell subsets to progression of pulmonary vascular wall remodeling or protection from its aggravation, they delineate challenges and chances of understanding inflammation as a crucial disease mediator of pulmonary hypertension.

Additional review articles focus on specific diseases. Hasselbach et al. summarize how limited inflammatory clearance after an insult, such as ischemia, viral infection or even surgical stress, can exacerbate heart failure. The COVID-19 pandemic has shown us how there is no “one size fits all” for therapy of inflammatory insults, especially not in patients with underlying cardiovascular disease.

In their systematic review of the preclinical evidence, Wortmann et al. highlight the importance of modulating inflammation as one of the few conservative treatment options of aortic disease. They analyze how targeting the NOD-, LRR- and pyrin domain-containing protein 3 (NLRP3) inflammasome can reduce aortic disease burden and associated complications, and may thus translate into clinical treatment options to prevent aortic wall destruction in humans.

By using an innovative stroke model in mice, Vornholz et al. demonstrated that right-sided stroke results in acute heart failure with reduced ejection fraction, bradycardia, hypotension, and elevation of cardiac troponin. In parallel, they observed a cardiac and systemic inflammatory response to stroke, with IL-6 and IL-1ß-dominated inflammation in the heart and increased cardiac and systemic numbers of granulocytes. Severity of cardiac alteration was associated with increased mortality. This study adds to the growing body of evidence that the heart is an end organ during cerebral injury.

In their review, Bello and colleagues highlight the role of Indoleamine-2,3-dioxygenase (IDO) in different immune-related cardiovascular diseases. They focus on its potential for decision-making and tailoring treatment strategies in autoimmunity and neurodegenerative disorders and address its role as a perioperative predictive biomarker in bacterial infection, influenza, sepsis, surgical trauma and in diseases related to oxidative stress.

A potential therapeutic approach is introduced by the group around Kumawat. They report anti-inflammatory effects of an anti-IL17A affibody on vascular smooth muscle cells and fibroblasts *in vitro*. In a mouse model of atherosclerosis, inflammation was reduced but burden of atherosclerotic plaque development remained unchanged.

The article collection spans the entire range from reviews summarizing the current knowledge in targeting innate and adaptive immunity, addressing specific diseases and describing predictive biomarkers to exploring therapeutic options to improve cardiovascular disease.

Research in innate and adaptive immunity in cardiovascular disease has proven that extensive studies on animal models can be translated into clinical trials, under careful consideration of who is in need of additional therapeutic options. Substantial progress has been made and several inflammatory targets have proven their central role for preventing cardiovascular disease and for treating its complications. However, this article collection also demonstrates that much more research effort is needed and that it is a long way to go in order to remove cardiovascular diseases from the top ranks on the WHOs list of leading causes of death.
